# Molecular and Genetic Aspects of Grain Number Determination in Rice (*Oryza sativa* L.)

**DOI:** 10.3390/ijms22020728

**Published:** 2021-01-13

**Authors:** Changxi Yin, Yanchun Zhu, Xuefei Li, Yongjun Lin

**Affiliations:** National Key Laboratory of Crop Genetic Improvement, Huazhong Agricultural University, Wuhan 430070, China; yinchangxi@mail.hzau.edu.cn (C.Y.); zhuyanchun@webmail.hzau.edu.cn (Y.Z.); lixuefei@webmail.hzau.edu.cn (X.L.)

**Keywords:** grain number per panicle, grain yield, phase transition, rachis branch, rice panicle, spikelet specialisation

## Abstract

Rice grain yield is a complex trait determined by three components: panicle number, grain number per panicle (GNPP) and grain weight. GNPP is the major contributor to grain yield and is crucial for its improvement. GNPP is determined by a series of physiological and biochemical steps, including inflorescence development, formation of rachis branches such as primary rachis branches and secondary rachis branches, and spikelet specialisation (lateral and terminal spikelets). The molecular genetic basis of GNPP determination is complex, and it is regulated by numerous interlinked genes. In this review, panicle development and the determination of GNPP is described briefly, and GNPP-related genes that influence its determination are categorised according to their regulatory mechanisms. We introduce genes related to rachis branch development and their regulation of GNPP, genes related to phase transition (from rachis branch meristem to spikelet meristem) and their regulation of GNPP, and genes related to spikelet specialisation and their regulation of GNPP. In addition, we describe other GNPP-related genes and their regulation of GNPP. Research on GNPP determination suggests that it is possible to cultivate rice varieties with higher grain yield by modifying GNPP-related genes.

## 1. Introduction

Rice is one of the most important food crops and feeds half of the world’s population [1,2]. Because of reductions in available land and the increasing global population, increasing the rice grain yield per unit area is crucial for food security, particularly in developing countries in Asia, such as in China and India [3].

Rice grain yield is primarily determined by three traits—grain number per panicle (GNPP), grain weight and number of panicles [4]. Because the rice grain yield per unit area is high, increasing the GNPP could further improve the grain yield [4,5,6]. Much research has been focused on GNPP determination, and considerable progress has been made in understanding the underlying regulatory mechanism. Here, we review progress in the molecular and genetic aspects of GNPP determination in rice.

## 2. Panicle Development and GNPP Determination in Rice

### 2.1. Panical Development

**Figure 1:**
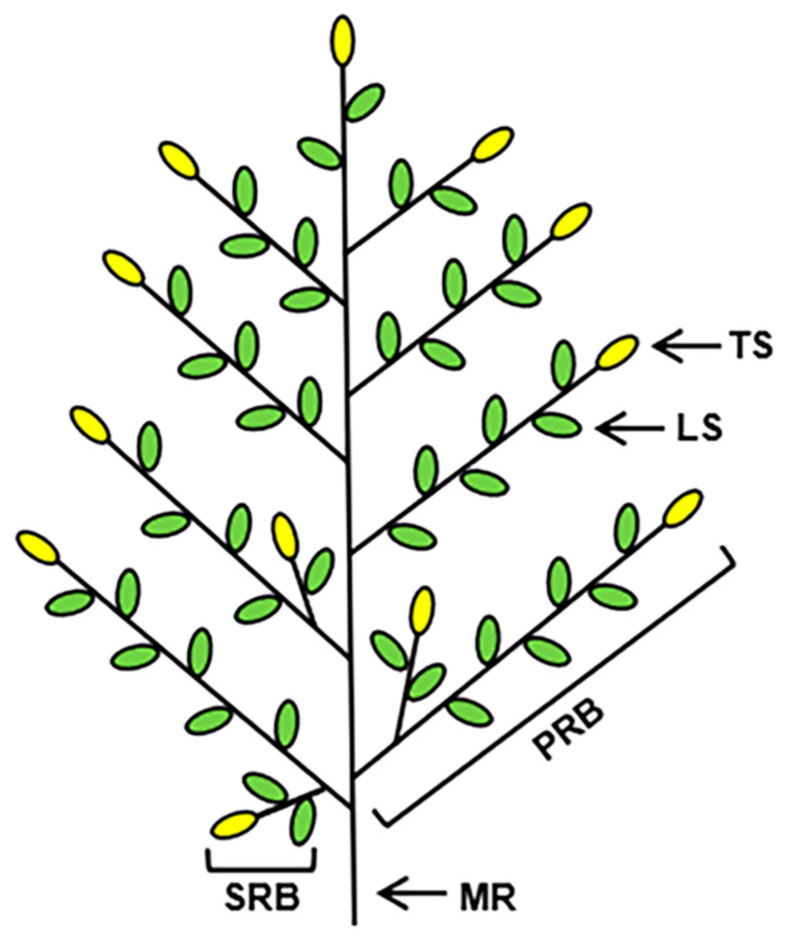
Panicle architecture of rice. The green ellipses show the lateral spikelets and the yellow ellipses show the terminal spikelets. LS, lateral spikelet; MR, main rachis; PRB, primary rachis branch; SRB, secondary rachis branch; TS, terminal spikelet.

The rice panicle is composed of the main rachis, rachis branches (primary rachis branches (PRBs) and secondary rachis branches (SRBs)) and spikelets (lateral and terminal spikelets) (Figure 1) [3,7]. In a few rice varieties, there are tertiary rachis branches in the panicle. GNPP determination involves the development of the inflorescence, formation of rachis branches and spikelet specialisation. During rice panicle development, the inflorescence meristem (IM) is an important regulator of GNPP formation [8]. In rice, transition to the reproductive phase involves the transformation of the shoot apical meristem (SAM) into the IM, initiating the growth of several lateral meristems as PRBs. Next, the IM loses its activity, leaving a vestige at the base of the uppermost PRB. The PRB meristem produces next-order branches as lateral meristems. The few initially formed lateral meristems grow as SRBs and later meristems directly from spikelet meristems. Lateral spikelets differentiate directly from newly formed lateral meristems, and terminal spikelets are converted from rachis branch meristems. Therefore, three factors—rachis branch formation, the transition from rachis branch meristem to spikelet meristem and spikelet specialisation—determine the overall architecture of the panicle and the GNPP in rice [9].

### 2.2. GNPP Determination in Rice

Plant hormones, such as auxin, gibberellin (GA), cytokinin (CK), abscisic acid (ABA) and ethylene, are involved in regulating panicle development and GNPP in rice [10,11]. Auxin has a pivotal role in panicle development, as it is required for the initiation and maintenance of axillary meristems. Auxin is produced mainly in growing shoot apices and is transported basipetally down the site along specific transport routes through polar transport machinery. Consequently, disruption in auxin synthesis or auxin transport results in fewer rachis branches and reduced GNPP in rice [12,13,14]. GA can affect panicle-associated traits including panicle length, rachis branch number and GNPP in rice [15]. A previous study demonstrated that *OsCYP71D8L* controls panicle-related traits by regulating GA homeostasis. Gain-of-function of *OsCYP71D8L* leads to shorter panicles, fewer rachis branches, and reduced GNPP in rice [16]. It has been reported that the fine-tuning of bioactive CK level in the IM is a critical trait for controlling the number of rachis branches and GNPP in rice. The decreased level of bioactive CK in rice IM is usually accompanied by fewer rachis branches and reduced GNPP [17,18], and the weakened CK signalling in rice IM also results in fewer rachis branches and reduced GNPP [19,20]. This evidence suggests that CK positively regulates GNPP in rice. On the other hand, the stress hormones such as ABA and ethylene negatively regulate the GNPP in rice [11,21]. In addition, signalling cascades and responses of several plant hormones overlap, and the molecular components are often shared among them. A complex network of effectors of multiple hormonal pathways collide and communicate to regulate critical agronomic traits including GNPP in rice [10].

Increasing evidence indicates that plant hormones mediate GNPP determination mainly through the transcriptional or post-transcriptional regulation of GNPP-related genes in rice [10,22,23]. Additionally, GNPP-related genes control panicle development mainly by regulating three factors, including rachis branch formation, the transition from rachis branch meristem to spikelet meristem and spikelet specialisation [9,17,18].

## 3. Functional Classification of GNPP-Related Genes and Their Regulation of GNPP

GNPP-related genes control the GNPP mainly by regulating rachis branch formation, the transition from the rachis branch meristem to the spikelet meristem and spikelet specialisation. To date, numerous genes that control GNPP by regulating rachis branch formation [24,25,26], and several that regulate the transition from the rachis branch meristem to the spikelet meristem [9,27,28,29,30], have been investigated. However, few genes involved in regulating spikelet specialisation have been reported [31,32,33]. Typically, inhibition of rachis branch meristem formation and acceleration of the conversion from the rachis branch meristem to the spikelet meristem reduce the GNPP by decreasing the number of rachis branches and vice versa [30,32,34]. In addition, the inhibition of spikelet specialisation may reduce the GNPP by inhibiting spikelet formation and vice versa [32,35].

### 3.1. Rachis Branch Development-Related Genes and Their Regulation of GNPP

#### 3.1.1. Positive Regulation of GNPP by Rachis Branch Development-Related Genes

*LAX PANICLE* (*LAX*) positively regulates the number of rachis branches and GNPP (Table 1). *LAX* encodes a plant-specific bHLH transcription factor. During rice panicle development, *LAX* is mainly expressed at the boundary region between the apical meristem and the newly formed lateral meristem, and it plays an important role in lateral meristem formation. Loss of function of *LAX* leads to a decreased number of rachis branches and a reduced GNPP. *LAX* cDNA is 1080 bp in length and contains one exon, encoding a protein of 215 amino acids. In the *lax*-*1* mutant, retrotransposon insertion causes a premature termination codon, and translation is terminated prematurely. In the *lax*-*2* mutant, there is a 36-kb deletion encompassing *LAX*. In the *lax*-*3* mutant, there is a 59-bp deletion in the bHLH domain of LAX. In the *lax*-*4* mutant, alanine 49 in the bHLH domain of LAX is changed to threonine. In the *lax*-*5* mutant, there is an arginine at position 50 of the bHLH domain of *LAX* [32]. Additionally, *lax*-*1*, *lax*-*4* and *lax*-*5* are *lax* mutants with mild changes in phenotype, with a reduced number of rachis branches, inhibited lateral spikelet development, normal terminal spikelet development and a reduced GNPP. By contrast, the panicles of severe *lax* mutants (*lax*-*2* and *lax*-*3*) have the main rachis but no rachis branches, and lateral spikelet development is completely blocked, whereas terminal spikelet development is inhibited [32]. Thus, the GNPP is reduced significantly in severe *lax* mutants. In a stronger allelic variant of the *lax* mutant, *lax1*-*2*, the initiation and maintenance of the rachis branch meristem, lateral spikelet meristem and terminal spikelet meristem are severely impaired. The transition from the partial rachis branch meristem to the spikelet meristem is delayed in the *lax1*-*2* mutant. The mild mutant *lax1*-*1* has no lateral spikelets but terminal spikelets and a small GNPP instead, whereas the severe mutant *lax1*-*2* has no spikelets and a few PRBs and SRBs [26].

*Ideal Plant Architecture 1* (*IPA1*)/*WEALTHY FARMER’S PANICLE* (*WFP*)/*SOUAMOSA PROMOTER BINDING PROTEIN-LIKE 14* (*OsSPL14*) positively regulates the number of rachis branches and the GNPP (Table 1). *IPA1*/*WFP*/*OsSPL14* encodes a SOUAMOSA PROMOTER BINDING PROTEIN-LIKE protein. Rice varieties with *IPA1*/*WFP*/*OsSPL14* have an ideal plant architecture, including more rachis branches and a greater GNPP. Overexpression of *OsSPL14* increases the number of rachis branches and the GNPP [36]. Moreover, OsmiR156 interacts with and lyses OsSPL14. As a result of a point mutation, *OsSPL14*^ipa1^ cannot be lysed by OsmiR156, resulting in a significant increase in the number of PRBs and SRBs and in the GNPP in panicles of *OsSPL14*^ipa1^ plants [37]. Therefore, *IPA1*/*WFP*/*OsSPL14* positively regulates the number of rachis branches and the GNPP, and upregulating *IPA1*/*WFP*/*OsSPL14* or enhancing the IPA1/WFP/OsSPL14 protein level increases the number of rachis branches and the GNPP, resulting in an increased grain yield [36,37].

*MONOCULM1*(*MOC1*) positively regulates tiller number, rachis branch number and GNPP in rice (Table 1). The *MOC1* cDNA is 1666 bp in length and contains four exons. *MOC1* encodes a GRAS-family nuclear protein consisting of 441 amino acids with a VHIID motif and an SH2-like domain. *MOC1* is expressed mainly in the axillary buds, initiating the buds and promoting their outgrowth. In *moc1*, a 1.9-kb retrotransposon is inserted at position 948, causing a premature translation stop, resulting in a truncated 338-amino-acid fusion protein with the last 22 residues encoded by the retrotransposon sequence. The *moc1* mutant has only a main culm without tillers as a result of a defect in tiller bud formation [24]. Moreover, *MOC1* regulates the GNPP mainly by controlling IM activity and bract primordium initiation in rice. Therefore, in *moc1* mutant plants, the number of PRBs and SRBs and the GNPP are decreased by the reduced IM activity and inhibited rachis primordium initiation [24,38]. These findings indicate that *MOC1* positively regulates the tiller number, number of rachis branches and GNPP in rice.

*Oryza sativa Homeobox1* (*OSH1*) positively regulates the number of rachis branches and the GNPP in rice (Table 1). *OSH1* cDNA is 1086 bp in length and contains five exons, encoding a protein product of 364 amino acids. *OSH1*, a rice homolog of *Knotted1-like Homeobox* (*KNOX*), a *KNOX* family class 1 homeobox gene, participates in SAM initiation and maintenance [39,40]. *OSH1* is self-induced and binds to five *KNOX* loci (*OSH1*, *OSH6*, *OSH15*, *OSH43* and *OSH71*), inducing their expression [22]. KNOX inhibits the expression of the GA biosynthesis gene *OsGA20ox* to reduce the GA content and promotes the expression of CK biosynthesis genes (including *OsIPT2* and *OsIPT3*) to increase the CK content [23]. KNOX induces SAM formation and maintains SAM activity by maintaining a high CK content and low GA content [22]. The self-induced expression of *OSH1* is necessary for SAM self-maintenance. Compared with the wild type, *osh1* mutant plants cannot maintain SAM activity, resulting in reduced numbers of PRBs and SRBs and a reduced GNPP [41].

*Small Panicle* (*SPA*) positively regulates the number of rachis branches and the GNPP (Table 1). *SPA* is functionally redundant with *LAX1* and regulates the rice axillary meristem. In *spa*, a *SPA* loss-of-function mutant, the rachis branches are short and abnormal, most base PRBs are missing and the numbers of SRBs and lateral spikelets are decreased significantly, resulting in a reduced number of rachis branches and a smaller GNPP. In a *spa* and *lax1-1* (a weak allelic mutant of *lax1*) double mutant, the panicle becomes a linear structure lacking all rachis branches. In addition, *spa* or *lax1* has little effect on tillering, which is almost completely inhibited in the *spa lax1* double mutant [32]. Therefore, *SPA* positively regulates the GNPP by promoting PRB, SRB and lateral spikelet formation.

*Short Panicle 1* (*SP1*) positively regulates panicle size, the length of the main rachis and rachis branches, the number of rachis branches and GNPP (Table 1). *SP1* encodes a putative transporter of the peptide transporter (PTR) family. SP1 contains a conserved PTR2 domain consisting of 12 transmembrane domains, and the SP1-GFP fusion protein was found to be localised in the plasma membrane. *SP1* is highly expressed in the phloem of the rachis branches of young panicles and may control panicle size by regulating rachis growth. SP1 has been suggested to be a nitrate transporter. However, transport of neither nitrate nor any other compound transported by PTR proteins has been detected, suggesting that SP1 requires other component(s) to function as a transporter, or that it transports unknown substrates [25]. Compared with the wild type, panicle elongation in *sp1* plants (loss of function of SP1) is defective, resulting in a shorter panicle. Moreover, the number of SRBs and the GNPP are significantly reduced as a result of the decreased number of PRBs [25]. Therefore, *SP1* positively regulates the GNPP by regulating the growth of the main rachis and rachis branches in rice.

*DENSE AND ERECT PANICLE1* (*DEP1*) positively regulates the number of rachis branches and the GNPP (Table 1). *DEP1* is located at a major quantitative trait locus (QTL) that controls rice grain yield. *DEP1*, a key gene with multiple functions including the control of rice grain yield, was isolated from Shennong 265, a super rice variety in northeast China. The dominant allele at the *DEP1* locus is a gain-of-function mutation causing truncation of a phosphatidylethanolamine-binding protein-like domain protein [42]. This allele increases meristem activity, increasing the number of rachis branches (PRBs and SRBs) and the GNPP, consequently increasing grain yield by 15–20% [42]. This allele is common to many high-yield rice varieties planted in a large area of China, suggesting that *DEP1* has played an important role in increasing rice grain yield in China.

*OsNAC2*/*OMTN2*/*Ostil1* positively regulates the panicle length, rachis branch number and GNPP (Table 1). *OsNAC2*/*OMTN2*/*Ostil1* encodes a NAC transcription factor. *OsNAC2* is located at the same locus as *OMTN2* and *Ostil1* [43,44,45]. The microRNA miR164b interacts with and lyses OsNAC2. Overexpressing OErN (mutation of OsNAC2), which is not lysed by miR164b, leads to increased panicle length, stem thickness and number of vascular bundles in stem and leaves, resulting in increased numbers of PRBs and SRBs and an increased GNPP and grain yield [46]. *IPA1* and *DEP1* are significantly upregulated in *OErN* plants, and regulation by *OsNAC2* of the number of rachis branches and the GNPP may be related to CK signalling. *OsNAC2-RNAi* plants, transgenic rice plants with low expression of *OsNAC2*, have a shorter panicle, thinner stem, decreased number of vascular bundles in the stem and leaf, decreased numbers of PRBs and SRBs, and a smaller GNPP and thus have a lower grain yield. In addition, overexpression of miR164b in the transgenic rice OE164 resulted in the fragmentation of OsNAC2. Compared with wild-type plants, OE164 had shorter panicles, lower GNPP and a lower grain yield [46].

*Grain Number4-1* (*GN4-1*) positively regulates the number of rachis branches, and *GN4-1* has a marked effect on the GNPP (Table 1) [47]. Insertion of a near-isogenic line (NIL; *GN4-1*) from Wuyunjing 8 into Zhonghui 8006 increases the number of PRBs and SRBs, the GNPP and the grain yield. Compared with Zhonghui 8006, the expression levels of *OsCKX2* (cytokinin oxidase) and other cytokinin oxidase genes (*OsCKX1*, *OsCKX4*, *OsCKX7*, *OsCKX8*, *OsCKX9*, *OsCKX10* and *OsCKX11*) were decreased significantly in NIL (*GN4-1*), elevating the contents of CKs (zeatin, zeatin riboside) and IM activity and increasing the number of PRBs and SRBs and the GNPP in NIL (*GN4-1*) [4]. *GN4-1* from Wuyunjing 8 promotes CK accumulation in rice inflorescences and increases the GNPP by 17% [4]. Therefore, *GN4-1* regulates the GNPP by controlling the CK content in rice inflorescences.

*Grain Number per Panicle1* (*GNP1*) positively regulates the number of SRBs and the GNPP (Table 1). A T-DNA insertion mutant (*gnp1-D*) with enhanced *GNP1* expression exhibits increased plant height, more rachis branches and a greater GNPP. Compared with the NIL-*GNP1*^LT^ (isogenic control) line, the total and solid grain numbers in the NIL-*GNP1*^TQ^ line increased by 56% and 28%, respectively. In the NIL-*GNP1*^TQ^ line, although there was no obvious increase in PRB number, the SRB number increased significantly, significantly increasing grain yield at multiple experimental sites in China [48]. *GNP1* may upregulate *KNOX* expression, inducing the expression of the CK biosynthesis gene *OsIPT*, and enhance IM activity by increasing the CK content and enhancing CK signalling. Moreover, *GNP1* reduces the contents of active GAs (GA_1_ and GA_3_) by promoting their inactivation via the upregulation of *GA2ox* expression, which may positively regulate SRB number and GNPP [48]. Therefore, *GNP1* may enhance IM activity by enhancing CK signalling and suppressing GA signalling, thus increasing the SRB number and GNPP [48].

*Grain Number per Panicle Gene4* (*Gnp4*)/*LAX PANICLE2* (*LAX2*) encodes a nuclear protein that regulates the formation of the axillary meristem and positively regulates the number of SRBs and the GNPP (Table 1). *Gnp4* is located within a 10.7-kb region in the long arm of rice chromosome 4. There is no sequence difference between the mutant and the wild type in this region, but there are differences in cytosine methylation levels in the CpG island region of the candidate gene promoter [49]. LAX2 is a nuclear protein with a plant-specific conserved domain that interacts with LAX1 and regulates axillary meristem formation in rice [50]. *Gnp4* is located at the same locus as *LAX2* [49,50]. The SRB of the *gnp4* mutant does not produce spikelets, and the SRB number and GNPP of the *gnp4 lax1-1* double mutant are significantly reduced [49]. The spikelets in the panicles of the *lax2* mutant are sparse, and the SRB number and GNPP of the *lax2-1* mutant are significantly reduced, although there is no difference in PRB number between *lax2-1* and wild-type plants. The decreases in rachis branch number and the GNPP are greater in the *lax1 lax2* double mutant than in either single mutant [50]. Therefore, *Gnp4*/*LAX2* positively regulates the number of SRBs and the GNPP in rice.

*PLANT ARCHITECTURE AND YIELD1* (*PAY1*) positively regulates the SRB number and the GNPP (Table 1). Zhao et al. constructed a mutant library by mutating YIL55 (infiltration system of wild-type rice) via EMS mutagenesis and investigated a mutant (*PAY1*) with an upright and compact architecture. YIL55 had a shorter plant height, more tillers, a larger tiller angle, a lower GNPP and lower grain yield [51]. By contrast, *PAY1* mutants had a taller plant height, fewer tillers, a smaller tiller angle, thicker culm, more SRBs, a larger GNPP and lower grain yield but exhibited no significant changes in PRB number. Auxin polar transport activity is weakened in *PAY1*, and so *PAY1* may regulate rice phenotype by influencing auxin polar transport and altering the distribution of endogenous indole-3-acetic acid (the most important auxin in higher plants) [51]. Compared with wild-type plants, *PAY1-OE* plants had a thicker culm, fewer tillers, taller plant height, no significant change in PRB number, more SRBs, a larger GNPP and a higher grain yield. By contrast, *PAY1-RNAi* plants had a thinner culm, more tillers, shorter plant height, fewer SRBs, a lower GNPP and a lower grain yield [51]. Therefore, *PAY1* positively regulates GNPP by increasing the number of SRBs in rice.

*LAX1*, *IPA1/WFP/OsSPL14*, *MOC1*, *OSH1*, *SPA*, *SP1*, *DEP1*, *OsNAC2* and *GN4-1* positively regulate the number of rachis branches and the GNPP. Loss of function of these genes reduces the number of PRBs and SRBs and the grain yield, whereas their upregulation or gain of function increases PRB and SRB numbers and the grain yield. By contrast, although *GNP1*, *GNP4*/*LAX2* and *PAY1* positively regulate the number of SRBs and the GNPP, they do not regulate the number of PRBs. However, the mechanisms by which these genes regulate the number of rachis branches and the GNPP are unclear, and some have not been cloned yet. Therefore, the positive regulation by these genes of the number of rachis branches and the GNPP warrants further investigation. It may be possible to increase the number of rachis branches and the GNPP using GNPP-related genes, thus improving grain yield.

#### 3.1.2. Negative Regulation of GNPP by Rachis Branch Development-Related Genes

*LARGER PANICLE* (*LP*)/*ERECT PANICLE3* (*EP3*) negatively regulates the number of rachis branches and the GNPP (Table 1). *LP* encodes an F-box protein rich in Kelch, and in situ hybridisation showed that *LP* is mainly expressed in the rachis primordium [52]. The F-box protein encoded by *EP3* may function as a subunit of E3 ubiquitin ligase in the recognition and degradation of specific substrates [53]. *EP3* and *LP* are located at the same locus [52,53,54]. LP interacts with SKP1-like protein to upregulate the expression of *OsCKX2* and decrease the CK level in rice inflorescence, leading to more PRBs and SRBs and a higher grain yield [52]. Compared to wild-type plants, the number of rachis branches, particularly of PRBs, increased significantly in an *lp* (loss of function of *LP*) mutant, increasing the GNPP and grain yield in *lp* mutant plants [52]. In addition, *lp* mutant plants are more lodging resistant compared to wild-type plants [52].

*DENSE AND ERECT PANICLE3* (*DEP3*)/*OspPLAIIIδ* negatively regulates the number of rachis branches and the GNPP (Table 1). *DEP3* and *OspPLAIIIδ* are located at the same locus [55,56]. Compared with wild-type *DEP3* gene, the *dep3* mutant allele loses 408 bp at LOC_Os06g46350, including the back 47 bp of the region encoding the third exon and the front 361 bp of the untranslated region (UTR) of the 3’-end [55]. The panicles of wild-type plants typically begin to droop after the flowering stage, but the panicles of *dep3* mutants remain upright from the flowering stage to the fully mature stage. Moreover, there are differences in the number of vascular bundles and the vascular bundle size, and in other phenotypic characteristics (including panicle length, rachis branch length and culm thickness) between *dep3* and wild-type plants. The *dep3* mutant plants have more vascular bundles in the upmost internode, a smaller vascular bundle, shorter panicle, shorter rachis branch and thicker culm [55]. In addition, compared with wild-type plants, *dep3* mutant plants have more PRBs and SRBs, a larger GNPP and a higher grain yield [55]. These results show that *DEP3* negatively regulates the number of rachis branches and the GNPP, thus increasing the grain yield.

*PANICLE PHYTOMER 2* (*PAP2*)/*OsMADS34* negatively regulates the number of rachis branches and the GNPP (Table 1). *PAP2* encodes an SEP-like MADS box transcription factor. PAP2 is expressed only in the inflorescence meristem, PRB meristem, lateral spikelet meristem, floret meristem, glumes and degenerated glumes during the panicle development stage [34]. The PRB number, SRB number and GNPP of a *pap2-1* (*PAP2* loss of function) mutant increased significantly compared with those of wild-type plants. The panicle size of *pap2-1* is slightly smaller than that of wild-type plants because panicle elongation in the mutant is inhibited, but plant height, tillering number and leaf number do not differ significantly between *pap2-1* and wild-type plants [34]. *OsMADS34*, also known as *PAP2*, is a specific SEP-like MADS box gene in gramineous plants that regulates panicle morphology by controlling the rachis number and GNPP [34,57,58]. *OsMADS34* is expressed in the root, stem, leaf, leaf sheath, panicle, glume and degenerated glume and strongly expressed in developing organs such as young panicles. OsMADS34 determines the grain size and rice grain yield. In *osmads34-t* mutants, the numbers of PRBs and SRBs are increased but the panicle length is reduced, and the GNPP is increased but the grain size and seed-setting rate are reduced [59]. Six MADS-box genes—*OsMADS50*, *OsMADS56*, *OsMADS22*, *OSMADS47*, *OsMADS55* and *OsMADS34*—have been detected in rice [60]. These MADS-box genes significantly increase the number of rachis branches, including PRBs and SRBs, by inhibiting the expression of *REDUCED CULM NUMBER 4*. In addition, knockout of *OsMADS50*, *OsMADS56*, *OsMADS22*, *OsMADS47* and *OsMADS55* in an *osmads34* mutant significantly increased the number of PRBs and SRBs and the GNPP [60]. These findings indicate that *PAP2*/*OsMADS34* negatively regulates the number of rachis branches and the GNPP in rice.

*Awn-1* (*An-1*) encodes a bHLH protein that positively regulates awn length and negatively regulates rachis branch number and GNPP in rice (Table 1). During spikelet development, *An-1* is first expressed in two degenerate glumes and two empty glume primordia, next in lemma primordia and palea primordia, and finally in stamens and carpel primordia. *An-1* expression gradually increases at the tip of the lemma primordium and is significantly enhanced in the awn primordium from the sixth to the eighth stage, gradually decreasing thereafter [61]. The *An-1* allele was introduced into Guanglu-Ai-4, an *indica* rice without awns, yielding the near-isogenic line NIL-*An-1*. The awn length and grain length increased, but the number of rachis branches and the GNPP decreased significantly in NIL-*An-1* compared with wild-type plants. Upregulation of *An-1* expression in inflorescences decreases IM activity, thus reducing the number of rachis branches and the GNPP. *An-1-OX*, a transgenic plant with high expression of *An-1*, has fewer PRBs and SRBs and a lower GNPP. By contrast, the number of rachis branches and the GNPP are increased in *An-1-RNAi* plants, in which *An-1* expression is knocked down [61].

*PROSTRATE GROWTH1* (*PROG1*) positively regulates prostate growth and negatively regulates the number of PRBs and SRBs and the GNPP in rice (Table 1). The semi-dominant gene *PROG1* is located between RM298 and RM481, the short-arm simple sequence repeat markers of chromosome 7 [62]. *PROG1* has been isolated and cloned by two research teams [62,63]. The *PROG1* cDNA is 833 bp long and encompasses a 486-bp open reading frame, a 147-bp 5’ UTR and a 200-bp 3’ UTR. *PROG1* encodes a 161-amino-acid Cys_2_-His_2_ zinc-finger protein, which is mainly expressed in the axillary meristem [62,64]. There is a base mutation in the coding region of the gene in cultivated rice that causes an amino acid substitution, which may be selected during artificial domestication [63]. During the evolution of rice, *PROG1* of wild rice evolved into *prog1* of cultivated rice, resulting in the loss of function of *PROG1*, which mediates not only the transition from prostrate growth to erect growth but also changes in panicle architecture—e.g., increasing the numbers of PRBs and SRBs, the GNPP and the grain yield of cultivated rice. One hundred eighty-two varieties of cultivated rice, including *indica* and *japonica* cultivars from 17 countries, carry identical mutations in the *prog1* coding region, suggesting that *prog1* has become fixed during artificial domestication in rice [62]. Therefore, *PROG1* negatively regulates the number of rachis branches and the GNPP in rice.

*DROUGHT AND SALT TOLERANCE* (*DST*) negatively regulates the CK content, reducing the number of rachis branches and the GNPP in rice (Table 1). DST is a zinc-finger transcription factor in rice that directly regulates the expression of the cytokinin oxidase-encoding gene *OsCKX2*, thus increasing the number of rachis branches, GNPP and grain yield [8,65]. DST is mainly expressed in the SAM, PRB, SRB and young spikelets of the developing panicle [8]. DST promotes the expression of *OsCKX2*, thus reducing the CK content, SAM activity, number of rachis branches and GNPP. DST is a transcription factor with a C_2_H_2_ zinc-finger domain, by which DST proteins bind to DST-binding sequence (DBS) elements [42]. DBS elements are present in the promoter region of *OsCKX2* and other *OsCKX* genes [8]. In the semi-dominant mutant *reg1*, a single base insertion in *DST* led to premature termination of protein translation, resulting in the loss of the transcriptional activation ability of DST, accompanied by decreased expression of *OsCKX2* and other *OsCKX* genes, an increased CK content in the IM, increased numbers of PRBs and SRBs, and an enhanced GNPP in rice [8].

*Grain Number2* (*GN2*) negatively regulates the number of rachis branches and the GNPP in rice (Table 1). Chen et al. inserted *GN2*, a gene from the wild rice Yuanjiang (*O. rufipogon* Griff.), into *indica* Teqing and obtained an introgression line (YIL9). Compared with Teqing, the GNPP, panicle length, PRB length and SRB number, but not the PRB number, of YIL9 were reduced [5]. Compared with wild-type plants, *GN2-OE*, a transgenic plant overexpressing *GN2*, had a reduced GNPP, shorter panicles and fewer SRBs [5]. Therefore, *GN2* negatively regulates grain yield mainly by reducing the number of SRBs and the GNPP in rice.

The above findings indicate that *LP*/*EP3*, *DEP3*, *PAP2*/*OsMADS34*, *An-1*, *PROG1* and *DST* negatively regulate the number of PRBs and SRBs and the GNPP in rice. Mutants with loss of function of these genes have more PRBs and SRBs and a larger GNPP compared to wild-type plants. *GN2* does not regulate the number of PRBs, but it negatively regulates the number of SRBs and the GNPP. However, the molecular mechanisms by which these genes regulate the formation of rachis branches and spikelets warrant further study. Elucidation of the molecular regulatory mechanisms of these genes will enable the breeding of rice varieties with more rachis branches, a larger GNPP and higher grain yield using molecular markers and gene editing.

### 3.2. Phase Transition (Rachis Branch Meristem to Spikelet Meristem)-Related Genes and Their Regulation of GNPP

*ABERRANT PANICLE ORGANIZATION 1* (*APO1*)/*SCM2* plays an important role during the phase transition from rachis branch meristem to spikelet meristem and positively regulates the number of rachis branches and the GNPP in rice (Table 1). *APO1*/*SCM2* contains two exons and encodes an F-box protein of 429 amino acids. *APO1* is mainly expressed in the SAM and lateral organ primordia. *APO1* regulates the timing of meristem transition, and loss of function of *APO1* resulted in premature spikelet formation and an extended period of lodicule and carpel formation in an *apo1* mutant [27]. In plants with *apo1-D*, a gain-of-function mutation of *APO1*, IM activity is prolonged, and conversion from the rachis branch meristem to the lateral spikelet meristem and from the rachis branch meristem to the terminal spikelet meristem is delayed, resulting in a larger IM, more PRBs and SRBs, and higher grain yield than in wild-type plants [9]. By contrast, in plants with *apo1*, a loss-of-function mutation of *APO1*, IM activity is shortened, resulting in a smaller IM, fewer PRBs and SRBs, and a decreased GNPP compared to wild-type plants [27]. In addition, the QTL containing *SCM2* controls culm thickness. Mapping cloning results showed that *SCM2* is equivalent to *APO1*, and that NILs carrying *SCM2* (NIL-*SCM2*) exhibited increased stem strength, more tillers and a larger GNPP, indicating that *SCM2* is pleiotropic. Although *SCM2* is a gain-of-function mutant of *APO1*, there are differences in panicle architecture, including the GNPP and spikelet shape, between *SCM2* and *APO1* plants [28]. Compared with wild-type plants, the number of rachis branches and the GNPP in NIL-*SCM2* plants are obviously increased. However, the GNPP of *APO1-OE* plants is not larger than that of wild-type plants, and the reason why overexpression of *APO1* does not increase the GNPP is unclear. Therefore, *APO1*/*SCM2* plays an important role in controlling the phase transition from rachis branch meristem to spikelet meristem as well as regulating the number of rachis branches and the GNPP in rice [28].

*RCN1* and *RCN2* increase the number of rachis branches and the GNPP by delaying the phase transition from rachis branch meristem to spikelet meristem (Table 1). *RCN1* and *RCN2* are rice TERMINAL FLOWER 1 /CENTRORADIALIS-like homologs, which regulate rice plant architecture (tiller number, plant height, panicle architecture) mainly by regulating meristem phase transition [29]. In *35S::RCN1* and *35S::RCN2* transgenic rice plants, phase transition to the reproductive stage is delayed, and transgenic rice plants have more tillers and denser panicles. Observation of the panicle structure revealed that the transition from the rachis branch meristem to the spikelet meristem is delayed, leading to the generation of higher-order rachis branches. Although there is no significant difference in PRB number between transgenic rice (*35S::RCN1* and *35S::RCN2*) plants and wild-type plants, the number of higher-order rachis branches, including SRBs and tertiary rachis branches, increases significantly, increasing the GNPP in *35S::RCN1* and *35S::RCN2* plants [29]. These findings indicate that *RCN1* and *RCN2* increase the number of rachis branches and the GNPP by delaying the phase transition to the spikelet meristem.

*TAWAWA1* (*TAW1*) positively regulates the number of rachis branches and the GNPP by regulating the phase transition of the meristem (Table 1). *TAW1* encodes a nuclear protein of unknown function and is highly expressed in the SAM, IM and rachis branch meristem. TAW1 regulates inflorescence development by extending IM activity and delaying the phase transition from rachis branch meristem to spikelet meristem [30]. In the dominant gain-of-function mutant *tawawa1-D*, IM activity is extended and spikelet specialisation is delayed, resulting in delayed IM abortion and prolonged rachis branch formation, thus increasing the number of rachis branches, including SRBs and terminal rachis branches, and the GNPP. By contrast, in *TAW1-RNAi* transgenic plants, the decreased TAW1 activity causes precocious IM abortion and spikelet formation, resulting in the generation of small inflorescences with reduced numbers of PRBs and SRBs and a decreased GNPP [30].

Therefore, *RCN1*, *RCN2* and *TAW1* positively regulate the number of rachis branches and the GNPP by regulating the phase transition from rachis branch meristem to spikelet meristem. Overexpression of these genes and their dominant gain-of-function mutation may delay the phase transition from rachis branch meristem to spikelet meristem and prolong the formation of higher-order rachis branches, including SRBs and terminal rachis branches, thus increasing the GNPP. In addition, compared with wild-type plants, the dominant gain-of-function mutant *apo1-D* and NIL-*SCM2* have more rachis branches and a larger GNPP. Therefore, rational use of these genes can enable the breeding of rice varieties with a large number of rachis branches, greater GNPP and high grain yield.

### 3.3. Spikelet-Specialisation-Related Genes and Their Regulation of GNPP

*FRIZZY PANICLE* (*FZP*)/*BFL1* positively regulates spikelet specialisation and GNPP (Table 1). *FZP* is a single-copy gene in rice, located in chromosome 7, that encodes an ERF transcription factor and is the rice ortholog of maize *BD1*. FZP is required to prevent the formation of an axillary meristem instead of a spikelet meristem and for the subsequent establishment of spikelet meristem identity [33]. In a *fzp* mutant, instead of proceeding to spikelet formation, axillary meristems are formed in the axils of rudimentary glumes and are either arrested or develop into higher-order rachis branches, such as SRBs and terminal branches. Therefore, although the *fzp* mutant has more SRBs and terminal rachis branches, it has fewer spikelets and a reduced GNPP. In addition, there is no significant difference in PRB number between *fzp-11* mutant and wild-type plants, but *fzp-11* mutants have more SRBs and a lower GNPP compared with wild-type plants [35]. Therefore, *FZP* may positively regulate the GNPP by promoting spikelet specialisation in rice. Additionally, *FZP* and *BFL1* are located at the same locus [66]. *BFL1* encodes a transcription factor with an EREBP/AP2 domain, and BFL1 is involved in mediating spikelet specialisation in rice. The *bfl1* mutant harbours a single *Ds* insertion in the upstream region of *BFL1*, and *Ds* insertion drastically reduces the *BFL1* transcript level in the *bfl1* mutant. Compared to the wild type, the *bfl1* mutant has a similar PRB number and more higher-order rachis branches but fewer spikelets and a reduced GNPP as a result of defective spikelet formation [66].

*FRIZZY PANICLE 2* (*FZP2*) positively regulates spikelet specialisation and GNPP (Table 1). *FZP2* plays an important role in spikelet specialisation. In *fzp2*, a loss-of-function mutant, rachis branch meristem activity is prolonged, and the phase transition from rachis branch meristem to spikelet meristem is delayed, significantly increasing the number of rachis branches and inhibiting lateral and terminal spikelet formation, resulting in a decreased GNPP in rice [31].

The above results indicate that *FZP*/*BFL1* and *FZP2* regulate GNPP by regulating spikelet specialisation. Compared with wild-type plants, although the number of higher-order rachis branches (PRBs and terminal rachis branches) increases, the number of spikelets and GNPP decrease in *fzp* and *fzp2*. Therefore, *FZP*/*BFL1* and *FZP2* play a key role in balancing the numbers of rachis branches and spikelets, and the regulation of these genes’ functions increases rice grain yield by balancing the number of rachis branches and spikelets.

## 4. Other GNPP-Related Genes and Their Regulation of GNPP

*Grain Number1a* (*GN1a*)/*Cytokinin Oxidase2* (*OsCKX2*) negatively regulate GNPP by reducing the CK content. The *Gn1a* locus is the main QTL affecting GNPP in rice. A QTL-*Gn1* was identified in the short arm of chromosome 1 using an *indica × japonica* cross (Habataki/Koshihikari). Further, 96 F2 plants produced by heterozygous NIL-*Gn1* (*Gn1*/*gn1*) plants were used to divide *Gn1* into two loci—*Gn1a* and *Gn1b*—with similar functions. *Gn1a* is located in the region (less than 2 cM) between R3192 and C12072S, and *Gn1b* is located upstream of *Gn1a* [17]. Additionally, using 13,000 F2 plants generated from hybrid plants of NIL-*Gn1a* (*Gn1a*/*gn1a*), *Gn1a* was confirmed to be located in the 6.3-kb region between 3A28 and 3A20, where there is only one open reading frame, namely *OsCKX2*. Complementary transformation experiments showed that *Gn1a* is *OsCKX2* [17]. Decreased expression or loss of function of *OsCKX2* increases the GNPP and grain yield of rice. *OsCKX2* is highly expressed in the leaf, stem, IM and spikelet, weakly expressed in the SAM and not expressed in the root or embryo. The SAM is responsible for the development of the aboveground organs, such as leaves, stems and flowers, after embryo transfer. CK plays a key role in maintaining SAM activity. Decreased *OsCKX2* expression results in the accumulation of CK in the IM, increasing the number of rachis branches and GNPP (Table 1), and ultimately increasing the grain yield [17].

*LONELY GUY* (*LOG*) positively regulates GNPP by promoting CK biosynthesis. *LOG* encodes a CK-activating enzyme that mediates the final step of CK biosynthesis. *LOG* is expressed at the apex of the SAM, indicating that CK activation occurs in a specific developmental region. CK promotes the development of the SAM. Loss of function of *LOG* results in premature termination of SAM activity, decreasing the number of rachis branches and GNPP. LOG may regulate SAM activity by controlling the concentration and spatial distribution of CK [18].

*Pyrabactin Resistance-Like* (*PYL*) positively regulates ABA signalling and negatively regulates GNPP in rice. PYLs are ABA receptors implicated in ABA signal transduction [11]. Mutations in ABA receptor genes can promote rice growth and increase grain yield [11]. Rice *PYL* genes are divided into two groups. Group I includes *PYL1*–*PYL6* and *PYL12*, and Group II includes *PYL7*–*PYL11* and *PYL13.* CRISPR/Cas9 has been used to edit *PYL* genes. Polygenic mutations in Group I can promote the growth of rice, but no such effect has been found for Group II. Compared with the wild type, a *pyL1*/*4*/*6* mutant exhibited enhanced growth and a higher grain yield in paddy fields with significantly increased panicle length, number of PRBs and SRBs, and GNPP [11]. Therefore, PYLs regulate rice panicle development, possibly in a manner dependent on the ABA signal transduction pathway.

*Grain Number, Plant Height, and Heading Date7* (*Ghd7*) is a major QTL that simultaneously controls the GNPP, plant height and heading stage of rice [67]. *Ghd7* encodes a nuclear protein with a CTT (CO-like and TIMING OF CAB1) domain [67]. Proteins containing this structural domain, such as *Arabidopsis CONSTANS* (*CO*) and rice *Hd1*, are implicated in the regulation of flowering time [68,69]. In the photoperiod-regulated flowering pathway, Ghd7 is located upstream of Ehd1 and Hd3a and acts via the Ghd7-Ehd1-Hd3A pathway. Ghd7 does not affect the expression of *Hd1*, but it does affect the expression of *Ehd1* and *Hd3a*. Under long-day conditions, *Ghd7* expression is upregulated, *Hd3a* expression is inhibited, and the heading date is delayed, enabling rice to make full use of light and temperature, thus increasing panicle length, plant height, GNPP and grain yield. In temperate regions, the short growth period of rice weakens or eliminates the function of Ghd7 in rice varieties in these regions, reducing or avoiding the effect of delayed heading date on rice grain yield. Therefore, the main function of *Ghd7* is to prolong the differentiation period of rice panicles and increase panicle length, thus enhancing the production of PRBs and SRBs and the GNPP as well as increasing the grain yield [67].

*Grain Number*, *Plant Height*, *and Heading Date8* (*Ghd8*)/*DTH8*/*OsHAP3H*/*LHD1* regulates GNPP by adjusting the photoperiodic pathway in rice. *Ghd8*, *DTH8*, *LHD1* and *OsHAP3H* are located at the same locus [70,71,72,73]. *Ghd8* encodes the HAP3 subunit of the HAP (heterotrimeric haem activator) complex [71]. *Ghd8* expression is maintained at a high level in meristems at all developmental stages under long-day conditions but is low under short-day conditions. Ghd8 has bidirectional regulatory effects on the Ehd1-Hd3a pathway. Under short-day conditions, Ghd8 upregulates *Ehd1*, *Hd3a* and *RFT1*, resulting in premature heading and flowering in rice. Under long-day conditions, Ghd8 inhibits the expression of these three genes and delays the heading and flowering dates of rice, thus increasing the number of rachis branches and GNPP. *Ghd8* may increase the number of tillers, PRBs and SRB as well as the GNPP of rice by upregulating the expression of *MOC1* [71].

*DTH7*/*Ghd7.1*/*OsPRR37* is a pleiotropic gene that controls heading date, plant height and GNPP in rice (Table 1). *DTH7*/*Ghd7.1*/*OsPRR37* encodes a pseudo-response regulator protein, and its expression is regulated by photoperiod. *OsPRR37*, *Ghd7.1* and *DTH7* are located at the same locus [74,75,76,77]. Under long-day conditions, DTH7 acts downstream of photosensitive pigment B and inhibits the expression of the anthocyanin genes *Hd3a* and *Ehd1* in rice, thus delaying flowering [76] and inducing an increase in the number of rachis branches, GNPP and grain yield per plant. *Ghd7.1* encodes pseudo-response REGULATOR37 (OsPRR37), which has a CCT domain [77]. *OsPRR37* is strongly expressed in the leaf and panicle, especially in the meristems of young panicles. Similar to *Ghd7*, under long-day conditions, *Ghd7.1* does not affect the expression of *Hd1*, but it does affect the expression of *Ehd1* and *Hd3a* [77]. In *indica* rice Zhenshan 97B, an eight-base deletion in *OsPRR37* leads to premature heading, shorter plant height, fewer rachis branches and a smaller GNPP [77]. Therefore, *DTH7*/*Ghd7.1*/*OsPRR37* positively regulates GNPP via the photoperiodic pathway in rice.

*GRAIN NUMBER*, *GRAIN LENGTH AND AWN DEVELOPMENT1* (*GAD1*) negatively regulates GNPP (Table 1). *GAD1*, located in the long arm of chromosome 8 of rice, encodes a cysteine-rich secretory peptide and has greater homology with the EPIDERMAL PATTERNING FACTOR-LIKE family of *Arabidopsis thaliana* [78]. The GAD1 protein has a signal peptide site at the N terminus, and the mature peptide has conserved cysteine residues at the C terminus. Common wild rice (W2014) has *GAD1*, but cultivated rice 93-11 harbours *gad1*. Wild rice typically has a lower GNPP, longer grains and long awns atop the grains. GAD1 may reduce the CK content by activating the expression of *DST* and *OsCKX2*, thus decreasing the GNPP of wild rice [78]. By contrast, a code-shifting mutation of *GAD1* in cultivated rice destroys the conserved cysteine structure, leading to loss of function of *GAD1*, thereby increasing the GNPP, shortening grain length and inhibiting awn development [78].

*NUMBER OF GRAINS 1* (*NOG1*) positively regulates the GNPP in rice (Table 1). In 2017, Chinese scientists cloned the *NOG1* gene, which is involved in the regulation of GNPP in rice. The introduction of *NOG1* increased grain yield by 25.8% in the *NOG1*-deficient rice cultivar Zhonghua 17, and overexpression of *NOG1* further increased grain yield by 19.5% in the *NOG1*-containing variety Teqing. *NOG1*, which encodes an enoyl-CoA hydratase/isomerase, increases the grain yield of rice by enhancing GNPP without reducing the number of panicles per plant or grain weight. Furthermore, *NOG1* plays important roles in regulating jasmonic acid homeostasis and the β-oxidation of fatty acids, which may be associated with its regulation of GNPP [79]. The mechanism by which *NOG1* regulates GNPP warrants further investigation.

## 5. Conclusions

GNPP, a grain-yield component in rice, is a hot research topic for breeders and molecular biologists. Several rice GNPP-related genes have been cloned, and their regulation of GNPP has been investigated. *LAX1*, *IPA1/WFP/OsSPL14*, *MOC1*, *OSH1*, *SPA*, *SP1*, *DEP1*, *OsNAC2* and *GN4-1* positively regulate the number of rachis branches and GNPP, whereas *GNP1*, *GNP4*/*LAX2* and *PAY1* positively regulate the number of SRBs and GNPP but not the number of PRBs (Table 1). By contrast, *LP*/*EP3*, *DEP3*, *PAP2*/*OsMADS34*, *An-1*, *PROG1* and *DST* negatively regulate the number of PRBs and SRBs and the GNPP in rice. *GN2* does not regulate the number of PRBs, but it negatively regulates the number of SRBs and the GNPP. Moreover, *RCN1*, *RCN2*, *TAW1* and *APO1*/*SCM2* positively regulate the number of rachis branches and the GNPP by regulating the phase transition from rachis branch meristem to spikelet meristem. In addition, *FZP*/*BFL1* and *FZP2* regulate the GNPP by regulating spikelet specialisation via balancing the numbers of rachis branches and spikelets (Table 1). Further, other GNPP-related genes and their regulation of GNPP have been reported (Table 1). However, the mechanisms by which some GNPP-related genes regulate GNPP determination are unclear in rice, and some have not been cloned yet. Therefore, the molecular regulatory networks of GNPP determination in rice need to be investigated. These molecular regulatory networks can be uncovered by constructing mutants and using molecular, genetic, physiological and -omics techniques, as well as bioinformatics. Furthermore, molecular marker-assisted selection, transgene techniques, gene editing and genome selection enable directional modification of GNPP-related genes and the polymerisation of favourable GNPP-related genes, allowing the cultivation of rice varieties with higher GNPP and grain yields.

## Figures and Tables

**Table 1:** GNPP-related genes and their regulation of GNPP. GNPP, grain number per panicle; Phase transition, from rachis branch meristem to spikelet meristem; PRB, primary rachis branch; SRB, secondary rachis branch; −, negative regulation; +, positive regulation.

Gene	RGAP Locus ID	Protein Products	PRB	SRB	Phase Transition	Spikelet Specialisation	Lateral Spikelet	Terminal Spikelet	GNPP	References
*LAX1*	LOC_Os01g61480	bHLH transcription factor	+	+	+		+	+	+	[26,32]
*IPA1*/*WFP*/*OsSPL14*	LOC_Os08g39890	SOUAMOSA PROMOTER BINDING PROTEIN-LIKE protein	+	+					+	[36,37]
*MOC1*	LOC_Os06g40780	GRAS-family nuclear protein	+	+					+	[24,38]
*OSH1*	LOC_Os03g51690	A protein product of 364 amino acids	+	+					+	[22,39,40,41]
*SPA*	Not reported	Small panicle	+	+			+		+	[32]
*SP1*	LOC_Os11g12740	A putative transporter of the peptide transporter (PTR) family	+	+					+	[25]
*DEP1*	LOC_Os09g26999	G protein gamma subunit	+	+					+	[42]
*OsNAC2*/*OMTN2*/*Ostil1*	LOC_Os04g38720	NAC transcription factor	+	+					+	[43,44,45,46]
*GN4-1*	Not reported	Grain number4-1	+	+					+	[4,47]
*GNP1*	LOC_Os03g63970	GA20-oxidase 1		+					+	[48]
*Gnp4*/*LAX2*	LOC_Os04g32510	A nuclear protein with a plant-specific conserved domain		+					+	[49,50]
*PAY1*	LOC_Os08g31470	Peptidase		+					+	[51]
*LP*/*EP3*	LOC_Os02g15950	F-box protein	−	−					−	[52,53,54]
*DEP3*/*OspPLAIIIδ*	LOC_Os06g46350	Patatin-related phospholipase A	−	−					−	[55,56]
*PAP2*/*OsMADS34*	LOC_Os03g54170	SEP-like MADS box transcription factor	−	−		+			−	[34,57,58,59,60]
*An-1*	LOC_Os04g28280	bHLH protein	−	−					−	[61]
*PROG1*	LOC_Os07g05900	A 161-amino-acid Cys_2_-His_2_ zinc-finger protein	−	−					−	[62,63,64]
*DST*	LOC_Os03g57240	Zinc-finger transcription factor	−	−					−	[8,65]
*GN2*	Not reported	Gain number2		−					−	[5]
*APO1*/*SCM2*	LOC_Os06g45460	F-box protein of 429 amino acids	+	+	−				+	[9,27,28]
*RCN1*	LOC_Os11g05470	ATP-binding cassette transporter		+	−				+	[29]
*RCN2*	LOC_Os02g32950	ATP-binding cassette transporter		+	−				+	[29]
*TAW1*	LOC_Os10g33780	Nuclear protein	+	+	−	−			+	[30]
*FZP*/*BFL1*	LOC_Os07g47330	ERF transcription factor		−	+	+			+	[33,35,66]
*FZP2*	Not reported	Frizzy panicle 2		−	+	+			+	[31]
*GN1a*/*OsCKX2*	LOC_Os01g10110	Cytokinin oxidase/dehydrogenase	−	−					−	[17]
*LOG*	LOC_Os01g40630	Cytokinin riboside 50-monophosphate phosphoribohydro-lase	+	+					+	[18]
*PYL1* *PYL4* *PYL6*	LOC_Os01g61210 LOC_Os03g18600 LOC_Os05g39580	ABA receptor protein	−	−					−	[11]
*Ghd7*	LOC_Os07g15770	CCT(CO, CO-LIKE and TIMING OF CAB1)	+	+					+	[67,68,69]
*Ghd8*/*DTH8*/*OsHAP3H*/*LHD1*	LOC_Os08g07740	HAP3 subunit of the HAP (heterotrimeric haem activator) complex	+	+					+	[70,71,72,73]
*DTH7*/*Ghd7.1*/*OsPRR37*	LOC_Os07g49460	A pseudo-response regulator protein	+	+					+	[74,75,76,77]
*GAD1*	LOC_Os08g37890	A cysteine-rich secretory peptide							+	[78]
*NOG1*	LOC_Os01g54860	Enoyl-CoA hydratase/isomerase							+	[79]

## Abbreviations

ABA	Abscisic acid
*An-1*	*Awn-1*
*APO1*	*ABERRANT PANICLE ORGANIZATION 1*
CK	Cytokinin
*DEP1*	*DENSE AND ERECT PANICLE1*
*DEP3*	*DENSE AND ERECT PANICLE3*
*DST*	*DROUGHT AND SALT TOLERANCE*
*EP3*	*ERECT PANICLE3*
*FZP*	*FRIZZY PANICLE*
*FZP2*	*FRIZZY PANICLE 2*
GA	Gibberellin
*GAD1*	*GRAIN NUMBER, GRAIN LENGTH AND AWN DEVELOPMENT1*
*Ghd7*	*Grain Number, Plant Height, and Heading Date7*
*Ghd8*	*Grain Number, Plant Height, and Heading Date8*
*GN1a*	*Grain Number1a*
*GN2*	*Grain Number2*
*GN4-1*	*Grain Number4-1*
*GNP1*	*Grain Number per Panicle1*
*Gnp4*	*Grain Number per Panicle Gene4*
GNPP	Grain number per panicle
HAP	Heterotrimeric haem activator
*LOG*	*LONELY GUY*
IM	Inflorescence meristem
*IPA1*	*Ideal Plant Architecture 1*
*KNOX*	*Knotted1-like Homeobox*
*LAX*	*LAX PANICLE*
*LAX2*	*LAX PANICLE2*
*LP*	*LARGER PANICLE*
*MOC1*	*MONOCULM1*
*NOG1*	*NUMBER OF GRAINS 1*
*OsCKX2*	*Cytokinin oxidase2*
*OSH1*	*Oryza sativa Homeobox1*
*PAP2*	*PANICLE PHYTOMER 2*
*PAY1*	*PLANT ARCHITECTURE AND YIELD1*
*PROG1*	*PROSTRATE GROWTH1*
PRR37	Pseudo-response REGULATOR37
PTR	Peptide transporter
*PYL*	*Pyrabactin Resistance-Like*
QTL	Quantitative trait locus
SAM	Shoot apical meristem
SRBs	Secondary rachis branches
*SP1*	*Short Panicle 1*
*SPA*	*Small Panicle*
*SPL14*	*SOUAMOSA PROMOTER BINDING PROTEIN-LIKE 14*
*TAW1*	*TAWAWA1*
UTR	Untranslated region
*WFP*	*WEALTHY FARMER’S PANICLE*
